# Genome-wide identification of tissue-specific long non-coding RNA in three farm animal species

**DOI:** 10.1186/s12864-018-5037-7

**Published:** 2018-09-18

**Authors:** Colin Kern, Ying Wang, James Chitwood, Ian Korf, Mary Delany, Hans Cheng, Juan F. Medrano, Alison L. Van Eenennaam, Catherine Ernst, Pablo Ross, Huaijun Zhou

**Affiliations:** 1 Department of Animal Science, University of California, Davis, Davis, CA USA; 2 Genome Center, University of California, Davis, Davis, CA USA; 3 USDA-ARS, Avian Disease and Oncology Laboratory, East Lansing, MI USA; 4 Department of Animal Science, Michigan State University, East Lansing, MI USA

**Keywords:** Long non-coding RNAs, Gene regulation, Epigenetics

## Abstract

**Background:**

Numerous long non-coding RNAs (lncRNAs) have been identified and their roles in gene regulation in humans, mice, and other model organisms studied; however, far less research has been focused on lncRNAs in farm animal species. While previous studies in chickens, cattle, and pigs identified lncRNAs in specific developmental stages or differentially expressed under specific conditions in a limited number of tissues, more comprehensive identification of lncRNAs in these species is needed. The goal of the FAANG Consortium (Functional Annotation of Animal Genomes) is to functionally annotate animal genomes, including the annotation of lncRNAs. As one of the FAANG pilot projects, lncRNAs were identified across eight tissues in two adult male biological replicates from chickens, cattle, and pigs.

**Results:**

Comprehensive lncRNA annotations for the chicken, cattle, and pig genomes were generated by utilizing RNA-seq from eight tissue types from two biological replicates per species at the adult developmental stage. A total of 9393 lncRNAs in chickens, 7235 lncRNAs in cattle, and 14,429 lncRNAs in pigs were identified. Including novel isoforms and lncRNAs from novel loci, 5288 novel lncRNAs were identified in chickens, 3732 in cattle, and 4870 in pigs. These transcripts match previously known patterns of lncRNAs, such as generally lower expression levels than mRNAs and higher tissue specificity. An analysis of lncRNA conservation across species identified a set of conserved lncRNAs with potential functions associated with chromatin structure and gene regulation. Tissue-specific lncRNAs were identified. Genes proximal to tissue-specific lncRNAs were enriched for GO terms associated with the tissue of origin, such as leukocyte activation in spleen.

**Conclusions:**

LncRNAs were identified in three important farm animal species using eight tissues from adult individuals. About half of the identified lncRNAs were not previously reported in the NCBI annotations for these species. While lncRNAs are less conserved than protein-coding genes, a set of positionally conserved lncRNAs were identified among chickens, cattle, and pigs with potential functions related to chromatin structure and gene regulation. Tissue-specific lncRNAs have potential regulatory functions on genes enriched for tissue-specific GO terms. Future work will include epigenetic data from ChIP-seq experiments to further refine these annotations.

**Electronic supplementary material:**

The online version of this article (10.1186/s12864-018-5037-7) contains supplementary material, which is available to authorized users.

## Background

Since the invention of genome sequencing technology, the focus of genomics has been to identify the genes present in an organism and understand their link to traits, or phenotypes, that the organism exhibits. As more is learned about genetics and the key role gene regulation plays in phenotypic expression, it is becoming clear that a complete understanding of the genome-to-phenome relationship will require a more comprehensive annotation of the genome than just protein-coding genes. RNA-seq data has revealed that while less than 5% of the human genome consists of protein coding sequences, most of the genome is transcribed [1–3]. Furthermore, comparative genome studies have shown evolutionary conservation in intergenic regions of the genome, indicating positive selection pressure and implying that these conserved regions have important functions [4–7].

One class of important regulatory elements that has recently been gaining attention is long non-coding RNAs (lncRNAs). These transcripts are distinct from miRNAs, snoRNAs, and others in that they are defined as greater than 200 bases in length and share some characteristics of mRNA, such as polyadenylation. LncRNAs were originally thought to not contain open reading frames (ORFs), however some have been found with short ORFs that may be translated, though the function of these is still a topic of debate [8, 9]. Some lncRNAs have been shown to have functions in regulating gene expression. *XIST*, for example, is a lncRNA that acts as one of the major components of the X-inactivation process in placental mammals [10]. *HOTAIR* is another lncRNA found on human chromosome 12. High expression of this lncRNA in breast cancer tumors is a significant predictor of metastasis [11]. HOTAIR is particularly notable as it was the first RNA discovered that is transcribed from one chromosome and regulates transcription of a gene on a different chromosome. Another lncRNA, *Malat1*, has been studied in mice and shown to affect the expression of neighboring genes on the same chromosome [12]. Long non-coding RNAs can therefore regulate genes in both *cis* and *trans*, demonstrating the importance of studying these molecules.

Many studies have identified genome-wide lncRNAs in model organisms such as human [13–18], mouse [18–22], zebrafish [23, 24], frog [25], fruit fly [26, 27], nematode [28], and *Arabidopsis* [29]. Some lncRNA identification efforts have focused on maize [30] and one of the primary malaria-causing parasite species, *Plasmodium falciparum* [31]. For farm animals, work has begun more recently to identify lncRNAs in chickens [32–37], cattle [38–43], pigs [33, 44–48], sheep [49–52], goats [53–56], and horses [57]. A recent review of lncRNA in livestock species provides a comprehensive overview of the current progress in the field [58]. Many of the lncRNA studies in livestock were performed using samples from varied developmental stages or using only one or two tissues while comparing between a control and experimental conditions. The chicken, cattle, and pig genomes are still lacking a comprehensive genome-wide catalog of lncRNAs in multiple tissues from adult animals.

The efforts of the ENCODE projects in creating comprehensive functional annotations of the human and mouse genomes have become a model for the Functional Annotation of Animal Genomes (FAANG) Consortium [59], whose goal is to functionally annotate all farm animal genomes. As one of the FAANG pilot projects, 48 tissue samples were collected from eight tissues across two biological replicates from chickens, cattle, and pigs. Adult male animals were used as they represent a transcriptionally stable state, avoiding the relatively more dynamic gene expression associated with development, growth, and the female reproductive cycle in certain tissues. Biological replicate animals were chosen to minimize biological diversity in each species. A highly inbred line was used for the chicken, the pigs sampled were littermates, and both cattle replicates had the same sire and were from a cattle line closely related to the cattle sequenced to construct the reference genome. The tissues were selected to include those that have a large number of associated quantitative phenotypic traits, focusing on traits relevant to the food production industry such as growth, health, feed efficiency, and disease resistance. The set of eight tissues used consisted of skeletal muscle, adipose, liver, lung, spleen, cerebellum, cortex, and hypothalamus.

As part of a FAANG pilot project, 48 stranded RNA-seq libraries were generated to identify lncRNAs in eight tissues from two biological replicates across the genomes of chicken, cattle, and pig. Using data from the same eight tissues in each species enabled the identification of tissue-specific lncRNAs, as well as those that appear to be generally expressed across the eight tissues examined. Finally, a comparative analysis of lncRNAs with shared expression between the three species was conducted to study evolutionary conservation of lncRNAs.

## Results

### Identification of lncRNAs

**Fig. 1:**
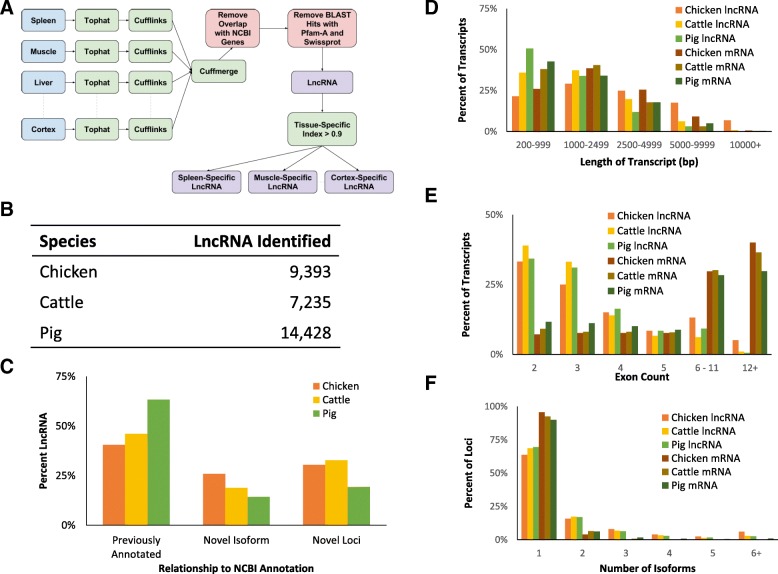
Identification of lncRNAs. **a** Computational pipeline used to identify lncRNAs. **b** Total number of lncRNAs identified per species. **c** The percentage of lncRNAs that match previously annotated lncRNAs in the NCBI annotation, are novel isoforms of previously annotated lncRNAs, or are expressed from unannotated genomic loci. A lncRNA was considered a novel isoform if it shared some exons with an annotated gene, but had additional unannotated exons or novel splicing. Previously annotated lncRNA had the same exons and splicing as an annotated gene. LncRNAs expressed from novel loci were in regions of the genome that no annotated transcript originated. **d** Distribution of transcript lengths of both lncRNAs and annotated protein-coding genes. **e** Distribution of the number of exons of both lncRNAs and protein-coding genes. **f** Distribution of the number of isoforms of both lncRNAs and protein-coding genes

Since lncRNAs are generally expressed at low levels [17] and can be hard to separate from noise in the data, the use of two biological replicates helped to verify the reproducibility of the results. Filtered and aligned RNA-seq reads (Table 1) for each of the eight tissues surpassed 100 million reads, a recommended threshold for identifying novel isoforms or transcripts that are expressed at low levels [60]. Table 2 and Table 3 show the number of genes and transcripts assembled for each RNA-seq library individually, which were then merged into a common transcriptome across all tissues. The number of transcripts in the merged transcriptome that were assigned each of the Cufflinks class codes, which indicate the relationship to previously annotated transcripts, are shown in Table 4. LncRNAs were identified by comparing them with known protein-coding genes in the NCBI annotations and with known proteins across any species in the Pfam [61] and Swiss-Prot [62] databases (Fig. 1a). A total of 31,057 lncRNAs were identified across chicken, cattle, and pig (Fig. 1b). The sequences are available in Additional files 1, 2 and 3 and their genomic locations and structures in Additional files 4, 5 and 6 Each lncRNA was placed into one of three categories based on the NCBI annotation for that species: previously annotated lncRNAs, novel isoforms of annotated lncRNAs, or transcripts from novel lncRNA loci (Fig. 1c, Table 5). On average, half of lncRNAs were previously annotated; however, a larger percentage of the lncRNAs from pig were previously annotated. In all three species, more novel lncRNAs are from novel loci rather than new isoforms of previously annotated lncRNAs. Including both novel isoforms and lncRNAs from novel loci, 5288 novel lncRNAs were identified in chickens, 3732 in cattle, and 4870 in pigs. LncRNAs were also compared to the NONCODEv5 database using sequence similarity [63]. Only 7.77% of predicted chicken lncRNAs and 5.57% of cattle lncRNAs had sequences similar to those in the NONCODE database, defined as having at least 50% sequence identity and the alignment covering at least 50% of the predicted lncRNA. In pigs, 37.59% of predicted lncRNAs were similar to those in the NONCODE database. These results are summarized in Table 6, and the individual lncRNAs with their matching NONCODE IDs are in Additional file 7.

**Table 1:** Total number of aligned and filtered RNA-seq reads per tissue

	Chicken	Cattle	Pig
Adipose	198,929,564	156,656,620	119,721,691
Cerebellum	242,807,223	246,658,282	152,762,359
Cortex	236,147,593	119,721,576	126,240,107
Hypothalamus	244,215,661	142,709,163	132,786,659
Liver	244,674,805	119,617,850	104,210,750
Lung	205,055,604	138,746,254	198,053,139
Muscle	238,435,618	140,106,635	155,724,909
Spleen	201,084,991	150,804,156	125,682,422

**Table 2:** The number of genes assembled from each RNA-seq library

	Chicken A	Chicken B	Cattle A	Cattle B	Pig A	Pig B
Adipose	25,837	27,020	50,396	51,271	49,322	47,401
Cerebellum	33,830	33,729	70,001	81,189	60,174	66,127
Cortex	35,110	35,984	46,410	52,946	50,951	51,532
Hypothalamus	33,437	34,457	53,784	54,949	53,811	46,592
Liver	25,127	27,235	45,275	47,518	43,793	44,592
Lung	30,680	29,747	50,051	59,447	66,299	61,041
Muscle	23,414	23,417	39,334	38,960	43,307	42,422
Spleen	30,927	31,752	56,125	62,107	61,337	57,744

**Table 3:** The number of transcripts assembled from each RNA-seq library

	Chicken A	Chicken B	Cattle A	Cattle B	Pig A	Pig B
Adipose	66,252	67,811	96,844	98,317	90,838	88,337
Cerebellum	76,797	76,515	119,305	131,204	104,161	110,994
Cortex	78,157	79,363	92,521	100,484	93,695	94,132
Hypothalamus	76,096	77,811	101,482	103,398	97,113	88,079
Liver	64,847	68,013	90,252	93,361	80,706	80,826
Lung	72,857	71,558	97,876	108,481	111,665	105,423
Muscle	61,921	61,825	82,076	81,887	82,664	81,214
Spleen	73,368	74,021	103,069	110,812	105,930	101,208

**Table 4:** The number of each Cufflinks “class code” in the transcriptome merged from all tissues

	=	j	u	x	o	s
Chicken	49,456	40,620	21,034	3205	802	0
Pig	54,311	41,237	35,046	4306	925	7
Cattle	64,413	45,759	30,504	3736	1071	0

“=” is a complete match of an existing transcript in the NCBI annotation. “j” is a potential novel isoform of an existing transcript. “u” is an unknown intergenic transcript. “x” is an exonic overlap on the opposite strand. “o” is an overlap with annotated exons, but is not classed as “j” because no splice sites match. “s” is an intronic overlap on the opposite strand. See http://cole-trapnell-lab.github.io/cufflinks/cuffcompare/ for more details

**Table 5:** The number of lncRNA transcripts and loci from NCBI annotations and this study

	Chicken	Cattle	Pig	Human	Mouse
NCBI Transcripts	6072	6187	14,503	27,986	21,705
Novel Transcripts	9393	7235	14,429	–	–
NCBI Loci	4167	4601	10,388	15,765	11,957
Novel Loci	4654	4325	8772	–	–

**Table 6:** LncRNA comparison with the NONCODEv5 database based on sequence similarity

	Novel LncRNA	NONCODE	Overlap
Chicken	9393	12,850	730
Pig	14,429	29,585	5424
Cattle	7235	23,515	403

While a coding potential score was not used for indentification of lncRNAs for this study, scores were calculated by FEELnc [64] that can be used as a confidence metric for further filtering of candidates. Using the default cutoff for calling a transcript coding or non-coding by FEELnc, 996 chicken lncRNAs, 475 pig lncRNAs, and 1326 cattle lncRNAs had scores predicting them as coding. This corresponded to 11.9, 3.4, and 22.4% of candidate lncRNAs respectively.

The number of exons, transcripts, and length of lncRNAs and mRNAs are shown in Fig. 1d-f. In all three species, the majority of mRNAs contain at least 5 exons, while most lncRNAs contain only 2 or 3 exons (see Fig. 1e), which is consistent with findings from the human ENCODE project [65]. Figure 1d shows the distribution of the lengths of lncRNAs and mRNAs, which were similar within each species. However, there were differences between species that are present in both lncRNAs and mRNAs. In pigs, about 50% of both types of RNA were in the 200–999 bp range, whereas only about 25% were in this range in chickens, and cattle were in-between. A general trend was observed where chicken transcripts of both types were generally longer than cattle and pig, while pig was the shortest.

### Potential regulatory targets of lncRNAs

**Fig. 2:**
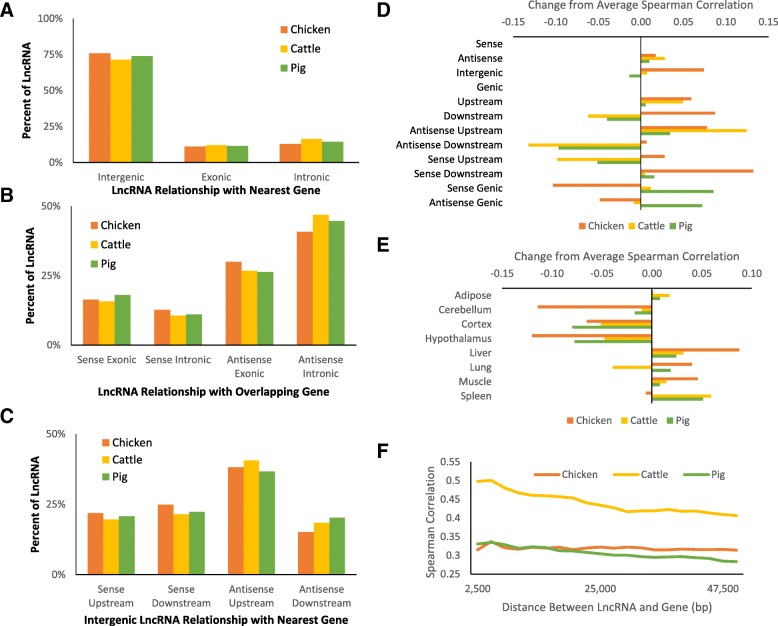
Potential regulatory targets of lncRNAs. **a** Percentage of lncRNAs that are intergenic, overlapping with exons of protein-coding genes, or overlapping with gene introns. LncRNAs were considered overlapping with exons if at least 1 base pair of a lncRNA exon overlapped a gene exon. A lncRNA was considered overlapping with gene introns if at least 1 base pair of a lncRNA exon overlapped a gene intron. Intergenic lncRNA had no exon overlap with any annotated protein coding gene region. **b** Percentage of genic (overlapping genes) lncRNAs that overlap on the same strand (sense) or opposite strand (antisense) and with exons or introns. **c** Percentage of intergenic lncRNAs that are upstream or downstream and on the same strand or opposite strand of the nearest gene. **d**, **e** Difference in the Spearman correlation of expression between lncRNA-mRNA pairs from the average correlation, grouped by positional relationship (**d**) and tissue (**e**). **f** Spearman correlation of expression of antisense upstream (divergent) lncRNA-mRNA pairs at different distances between the transcripts

To analyze potential regulatory function, each lncRNA was paired with the nearest protein-coding gene as a potential regulator of that gene. If no gene was within 50 kb upstream or downstream of a lncRNA (in other words, the distance between the transcribed regions), that lncRNA was not included in this analysis. Excluded lncRNAs represented 12.9% of lncRNAs in chickens, 16.8% of lncRNAs in cattle, and 21.5% of lncRNAs in pigs. Over 90% of all three genomes are distally intergenic enough to exclude any lncRNA by the above criteria, yet not even a quarter of lncRNAs were found in these regions. This reinforces the potential regulatory roles that lncRNAs may have on genes. The remaining lncRNAs were then labeled as intergenic if they did not overlap the annotated gene region, exonic if they overlapped an exon by at least 1 bp, and intronic if they overlapped only introns (Fig. 2a). The exonic and intronic lncRNAs were then categorized based on whether they were on the same strand (sense) or opposite strand (antisense) of the protein-coding gene (Fig. 2b), while the intergenic lncRNAs were categorized by strand and by whether they were upstream or downstream based on transcriptional direction of the coding gene (Fig. 2c). Table 7 shows in detail the number of lncRNAs in each of these groups. Many exon-overlapping lncRNAs overlapped only small portions of exons. Other lncRNA exons overlapped a full protein-coding exon, but contain novel exons that do not appear to be part of an annotated gene. Regardless of the nature of the overlap, the resulting lncRNA does not have any similarity to known protein-coding transcripts or exhibit similarity to any known protein domain, and therefore may be a non-coding isoform of the gene.

**Table 7:** Number of lncRNAs in each genomic location group

	Chicken	Cattle	Pig
Sense Intergenic Upstream	1302	843	1733
Sense Intergenic Downstream	1679	923	1868
Antisense Intergenic Upstream	2063	1747	3069
Antisense Intergenic Downstream	1168	790	1696
Intergenic, No Gene Within 100 kb	1208	1216	3109
Sense Containing Exonic	227	182	344
Sense Overlapping Exonic	48	46	79
Sense Nested Exonic	49	41	109
Sense Containing Intronic	58	30	72
Sense Overlapping Intronic	27	25	21
Sense Nested Intronic	166	128	232
Antisense Containing Exonic	8	12	14
Antisense Overlapping Exonic	465	372	565
Antisense Nested Exonic	119	75	198
Antisense Containing Intronic	110	97	205
Antisense Overlapping Intronic	362	418	622
Antisense Nested Intronic	334	290	493
Total	9393	7235	14,429

In all three species, about 25% of the lncRNAs that were included in this analysis overlap the genic region, with the other 75% divided evenly between upstream or downstream location relative to the protein-coding gene. While the lncRNAs within the downstream region of genes did not appear to have any strand correlation with the gene (they were equally sense or antisense), there was a higher prevalence of antisense lncRNAs within the upstream region of genes in all three species. The Spearman correlation of the expression of the lncRNAs with their nearest genes was used to provide evidence for potential *cis-*regulatory function. To compare this correlation between groups and species, the average correlation was calculated for each species, then the difference was calculated from this average for each group of lncRNAs based on their positional relationship with the nearby gene, e.g. antisense upstream (Fig. 2d), and also for each tissue (Fig. 2e). A higher correlation between the expression of upstream antisense lncRNA-gene pairs was observed across all three species, supporting the potential co-regulation of these transcripts. The correlation in expression of intergenic lncRNA gene pairs was generally higher in cattle compared to chicken and pig, however in chicken the correlation was not affected by the distance of the lncRNA from the gene, while in cattle and pig shorter distances are associated with higher correlation (Fig. 2f). The lncRNA-gene pairs and their positional relationships are available as Additional files 8, 9 and 10, and the expression for every lncRNA in each sample is shown in Additional files 11, 12 and 13.

### Tissue-specific lncRNAs

**Fig. 3:**
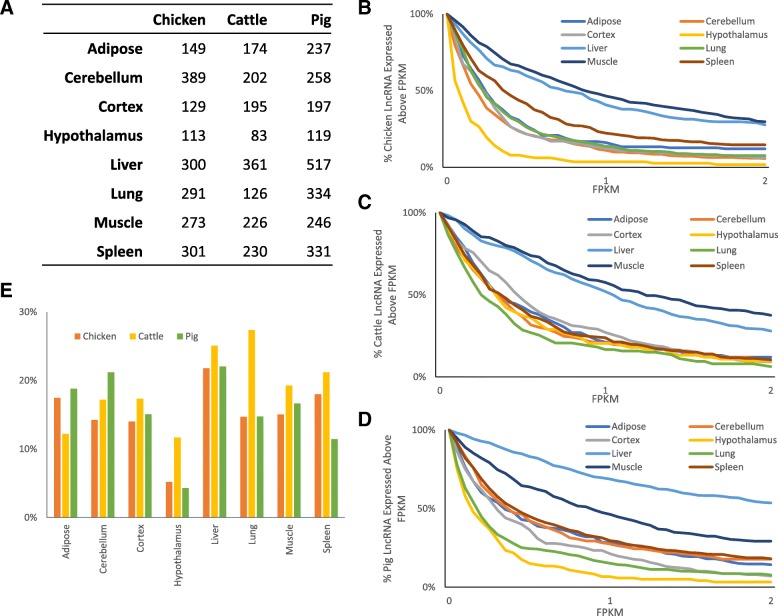
Tissue-specific lncRNAs. **a** The number of tissue-specific lncRNAs identified per species and tissue. **b**, **c**, **d** The percentage of tissue-specific lncRNAs expressed above various FPKM levels in chicken (**b**), cattle (**c**), and pig (**d**) respectively. **e** The percentage of protein-coding genes associated with tissue-specific lncRNA that are also tissue specific

Tissue-specific lncRNAs were identified using a Tissue Specific Index (see Methods). Fewer tissue-specific lncRNAs were seen in brain and adipose across the three species (Fig. 3a). As lncRNAs are known to be expressed at lower levels than mRNAs [17], any cutoff would be arbitrary, therefore lncRNAs that were expressed at any non-zero level were included. The percentage of lncRNAs expressed at or above a sliding cutoff was graphed, and in all three species lncRNAs specific to liver and muscle stood out as being expressed at higher levels than other tissues (Fig. 3b-d). The Tissue Specific Index calculated for each lncRNA is shown in Additional files 14, 15 and 16. The same analysis was repeated, but instead by calculating tissue-specificity using the expression of lncRNA loci rather than the expression of individual transcripts. In other words, the expression of multiple transcripts originating from the same loci would have been measured by a single expression value. The results mirrored the trends of the transcript-level analysis and are not presented in detail.

The gene ontology (GO) terms enriched in the set of genes associated with nearby tissue-specific lncRNAs were analyzed to understand the potential regulatory function of these lncRNAs (Additional files 17, 18 and 19). The tissue-specific index was calculated for these sets of associated protein-coding genes, and the percentage found to be tissue-specific is shown in Fig. 3e. On average across all species and tissues, only 17% of these genes were tissue-specific, with a maximum of 27% in cattle liver (Fig. 3e). Only two tissues had GO terms that were enriched across all three species. In cerebellum, nervous system development, generation of neurons, positive regulation of developmental process, regulation of cell differentiation, and regulation of multicellular organismal development were enriched in chicken, cattle, and pig. In cortex, nervous system development was enriched in all three species. While no other GO terms were enriched across all three species in the same tissue, related GO terms were enriched across species in some tissues, or GO terms were shared between two species. In adipose, skeletal system development was enriched in both cattle and chickens. GO terms related to the skeletal system did not appear in adipose from pigs. In addition to the GO terms shared across all species previously reported, some brain tissues contained GO terms specific to individual brain regions. Regulation of circadian rhythm was enriched by lncRNAs specific to the hypothalamus in chickens, and spinal cord development was enriched by lncRNAs specific to the cerebellum in cattle. GO terms associated with vasculature were enriched in the cerebellum and hypothalamus chicken: circulatory system development in hypothalamus, blood vessel morphogenesis in cerebellum. In liver, many metabolic process related GO terms were enriched for cattle and pig such as monocarboxylic acid metabolic process in cattle and alcohol metabolic process in pig; however, these were absent in chickens. No GO terms were significantly enriched for lung in chickens, but in cattle and pigs significantly enriched GO terms included lung morphogenesis and immune response in pigs and cardiovascular system development in cattle. For muscle, very few terms were significantly enriched in cattle, but muscle tissue development was the most significant. Heart morphogenesis was the most significantly enriched term for muscle in pigs, which only had a total of three significantly enriched GO terms. Chicken had comparatively more significantly enriched terms in muscle, including skeletal muscle development. Finally, lymphocyte or T cell activation were enriched GO terms for spleen in all three species.

### Conservation of lncRNAs

**Fig. 4:**
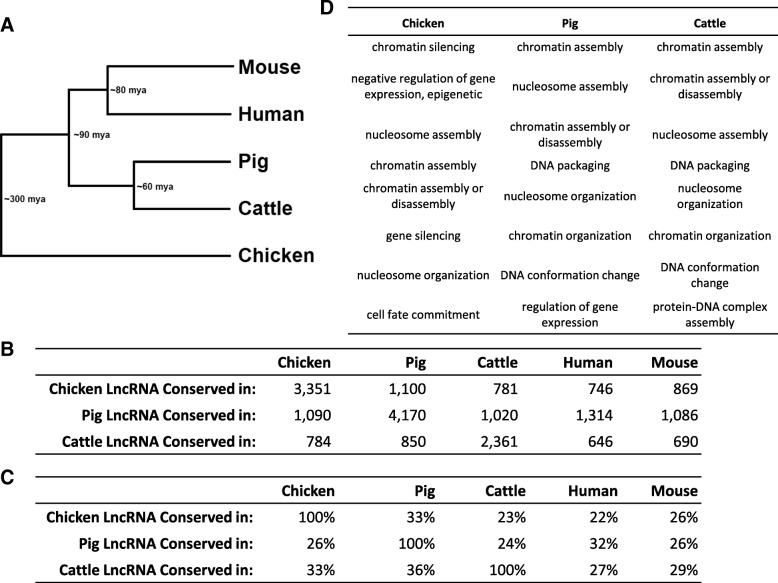
Conservation of lncRNAs. **a** Phylogenetic tree of the five animal species used for conservation analysis. **b** LncRNAs positionally conserved in other species. The numbers with the same species on the row and column indicate lncRNAs that are within 50 kb of protein-coding genes with orthologs in the other four species. Because the analysis relied on associating lncRNAs with genes that had orthologs in the other species, this number represents the number of lncRNAs that were included in the conservation analysis. **c** The percentage of lncRNAs positionally conserved in other species. **d** The top 8 GO terms, ranked by lowest FDR, enriched in lncRNAs conserved across all five species

The lncRNAs identified in this study were used to analyze the evolutionary conservation of lncRNAs. In addition to chicken, cattle, and pig, the annotated lncRNAs from human and mouse were included. As the only non-mammal, chicken is the most evolutionarily distant of the species, while cattle and pig are more closely related to each other than to human or mouse (Fig. 4a). Previous studies have shown that lncRNAs are not well conserved at the sequence level [66]. Therefore, positional conservation was analyzed. Using the lncRNA-gene pairs used in the previous analysis (Fig. 2), a lncRNA from one species was considered conserved in another species if the genes paired to each lncRNA were orthologs of each other. There was ~ 30% conservation in all species (Fig. 4b, c). A total of 39 ortholog groups were identified containing lncRNAs across the five species, consisting of 64 chicken lncRNAs, 55 cattle lncRNAs, 67 pig lncRNAs, 78 mouse lncRNAs, and 113 human lncRNAs. These lncRNAs are listed with their associated genes in Additional file 20. A GO term analysis of the genes associated with conserved lncRNAs showed that they have functions fundamental to cell biology (Fig. 4d). Chromatin assembly and nucleosome organization appeared in all three farm animal species along with related terms. Multiple sequence alignments performed on each of the groups of lncRNAs (Additional file 21) showed some regions of conservation between the species, although not at the magnitude of what would be expected of orthologous protein-coding genes.

## Discussion

The major goal of this study was to identify tissue-specific lncRNAs, evolutionarily conserved lncRNAs, and their potential regulatory functions across three farm animal genomes using deep RNA sequencing from eight tissues and two biological replicates. A major strength of this study compared to other lncRNA identification studies was the consistency in the methods used to obtain the data across the tissues and species. Because all the data were generated in the same lab by the same personnel and followed the same procedure from the same eight tissues taken from adult males, a comparison of lncRNAs among the three species with limited potential confounding factors such as different developmental stages, tissue types, or sexes was performed. Such a comparison would not have been possible using existing lncRNA annotations from Ensembl or NCBI, or by leveraging lncRNA sets previously identified by other researchers.

### Identification of lncRNAs

The observation that mRNAs contain on average more exons than lncRNAs is consistent with findings from the human ENCODE project [65]. However, no large difference was observed in the length of lncRNAs compared to mRNAs, despite the difference in exon count. This indicates that the exons in lncRNAs were generally larger than in mRNAs. Interestingly, a relatively large percentage of chicken lncRNAs were over 10,000 bp long when compared to both the lncRNAs of cattle and pig, and the mRNAs across all three species. Given the higher depth of RNA-seq achieved compared to the other two species (see Table 1), and the smaller size of the chicken genome (one third that of mammals), this observation may suggest that lncRNA transcripts in close proximity to one another in the genome may be combining during transcript assembly, or un-spliced transcripts may be causing introns to be occasionally sequenced and included in the assembly. In addition, while the majority of both lncRNAs and mRNAs only had a single isoform, this was more pronounced in mRNAs where at least 90% of genes had a single isoform in all species. This is contrary to the results from the ENCODE projects, where lncRNAs had generally fewer isoforms than mRNAs [65]. We speculate that the difference between this study and ENCODE might be an artifact of the transcript assembly and merging process, as many lncRNA isoforms differ only in exon length, not count, and are candidates for merging into a single isoform.

The proportion of lncRNAs categorized into each positional relationship to nearby protein-coding genes was very similar between species, as shown in Fig. 2a-c. However, the percentage of lncRNAs not categorized due to being outside the 50 kb window of any gene was lowest in chickens, as expected due to their small genome. The chicken genome is roughly one third the size of mammalian genomes, but with a similar number of genes. While the chicken has the lowest rate of excluded lncRNAs, there was still a notable difference between cattle and pig. The quality of the reference genomes and annotations for these species are being continually improved, and so a difference of quality in the current genomes could be causing this disparity.

Across all species, intergenic lncRNAs that were antisense to the nearest protein coding gene showed a prevalence for being upstream of those genes, while lncRNAs that were on the same strand as the nearest protein coding gene were equally upstream and downstream. Because the transcripts are on opposite strands and upstream of each other, they may share a promoter region if they are close enough. This sharing of regulatory regions could allow co-evolution of lncRNA and gene, leading to a higher prevalence of this upstream antisense relationship.

### Tissue-specific lncRNAs

Tissue-specific lncRNAs were identified, resulting in a few hundred per tissue per species (Fig. 3a). The potential function of these lncRNAs was predicted by examining GO term enrichment of the nearest protein-coding genes. For many tissues, terms with highly significant enrichment were associated with functions fundamental to those tissues, which has been seen in previous studies of mammalian lncRNAs [67]. Immune system terms, and more specifically lymphocyte activation, were enriched in spleen in all three species, with chicken GO term enrichment even more specific with T cell activation, which suggests expression of these spleen-specific lncRNAs are important for immune function. GO terms related to circulatory system were prevalent in tissues with a high density of blood vessels. This prevalence was observed across the three species in lung and brain, and in spleen from pigs and chickens. Less than 20% of genes associated with tissue-specific lncRNAs were themselves tissue-specific in their expression (Fig. 3e). This is not surprising, as studies looking at the regulatory mechanisms of specific lncRNAs have found both positive and negative regulatory functions, including post-transcriptional regulation [68]. When performing this analysis, an unadjusted *p*-value of 0.01 was used as a significance cutoff, rather than a value adjusted for multiple testing such as false discovery rate (FDR). This choice was made because the assumption that a lncRNA regulates the nearest protein-coding gene is a useful heuristic, but likely produces some false positives which should be considered when interpreting these results. The use of a more relaxed statistical significance cutoff yielded many of the biologically interesting results which would not have been seen using FDR. Unfortunately, few options exist currently to predict the regulatory target of lncRNAs.

### Conservation of lncRNAs

One of the main goals of this study was to identify the conservation of lncRNAs across three evolutionarily diverse species. Previous studies have found few conserved sequences across the lncRNAs among different organisms, even among closely related species [66]. Therefore, conservation analysis across species based on synteny was proposed. LncRNAs from the human and mouse NCBI annotations were also included so the conservation across five species could be analyzed. Because the human and mouse data do not have complete consistency in tissue, developmental stage, and sex from the data generated for this study, it was only appropriate to examine the conservation of chicken, cattle, and pig lncRNAs in mouse and human, but not vice versa. While a greater conservation was expected among the four mammalian species than with chicken, this was not reflected in this study’s results. This may simply be due to differences in the number of identified lncRNAs, which depends on the reference genome annotation quality. However, it may also suggest that most lncRNAs evolved very quickly and are not well conserved, with a small group of conserved lncRNAs representing evolutionarily ancient sequences. Such a hypothesis is supported by the 39 groups of orthologs that contain a lncRNA from all five species. The GO term analysis of nearby genes yielded biological processes that are common to cells across all eukaryotes, and would therefore be conserved over long evolutionary distances. These lncRNAs have been conserved for at least 300 million years, when the ancestors of birds and mammals diverged, and may be much older.

## Conclusions

This study identified 9393 lncRNA transcripts from 4654 loci in chickens, 7235 lncRNAs from 4325 loci in cattle, and 14,429 lncRNAs from 8772 loci in pigs. About half of these lncRNAs were previously annotated in the NCBI annotations of these species, with the remaining half consisting of approximately 50% novel transcripts of previously annotated lncRNAs and 50% lncRNAs identified at loci from which no currently annotated transcript originates.

Synteny-based conservation analysis across five evolutionarily diverse species (farm animals plus mouse and human) revealed a total of 39 distinct groups of lncRNAs. Conserved lncRNAs were associated with coding genes involved in epigenetic regulation and the physical structure of DNA (Fig. 4d).

Tissue-specific lncRNA analysis indicated that a greater proportion of lncRNAs specific to muscle and liver were highly expressed compared to the six other tissues. GO terms of coding genes associated with tissue-specific lncRNAs were enriched for tissue-specific functions. For example, in all three farm animal species, GO terms enriched in spleen were associated with lymphocyte activation and other immune-related GO terms.

This initial analysis revealed many novel insights into potential regulatory roles for lncRNAs with regard to tissue specificity and evolutionary conservation. As a part of ongoing FAANG research, ChIP-seq is being employed using the same tissue samples from this study to profile four histone modifications (H3K4me3, H3K27me3, H3K4me1, and H3K27ac) associated with promoters and enhancers, as well as binding sites for the transcription factor CTCF to identify insulators. This will further our understanding of the epigenetic regulation of protein-coding genes by lncRNAs. Additionally, ISO-seq, for full transcript sequencing, and RAMPAGE [69], for the accurate detection of transcription start sites, efforts are also underway, which will further refine the accuracy of these lncRNA annotations.

## Methods

### Genetic resources

Tissues were collected specifically for this study with all necessary permissions granted, following Protocol for Animal Care and Use #18464, approved by the Institutional Animal Care and Use Committee (IACUC), University of California, Davis. Animals were euthanized for collection of tissues from adipose, cerebellum, cortex, hypothalamus, liver, lung, skeletal muscle, and spleen and flash frozen in liquid nitrogen, then stored at − 80 °C until processing. Chickens were euthanized using CO2 under USDA inspection and samples were collected from two male F1 crosses of Line 6 and Line 7 from the Avian Disease and Oncology Laboratory (ADOL) at 20 weeks of age. Cattle were slaughtered by captive bolt under USDA inspection and samples were collected at University of California, Davis, from two intact male Line 1 Herefords provided by Fort Keogh Livestock and Range Research Lab at 14 months of age. Both individuals shared the same sire. Pigs were humanely slaughtered under USDA inspection and samples were collected from two castrated male littermate Yorkshires at Michigan State University at 6 months of age. The ages for all animals correspond with the sexually mature adult stage for their species.

### Library preparation and sequencing

Total RNA was isolated using Trizol (Invitrogen, Carlsbad, CA) according to the manufacturer’s protocol. DNase I (Ambion, Austin, TX) digestion was carried out after RNA isolation and the RNA concentration and purity were determined by measuring absorbance at 260 nm and A260/A280 ratio using a NanoDrop ND-1000 spectrophotometer (NanoDrop Technologies, Wilmington, DE). RNA samples were stored at − 80 °C until further use. Total RNA (1 μg) was subjected to two rounds of hybridization to oligo (dT) beads (Invitrogen, Carlsbad, CA) to enrich poly-adenylated transcripts. Stranded RNA-seq libraries were prepared using the TruSeq RNA Illumina protocol, and libraries were sequenced on an Illumina HiSeq-3000 using 100 bp PE to a depth of at least 50 million reads per library, or 100 million reads per tissue (when replicates were combined).

### Read mapping and transcript assembly

Reads were trimmed to remove adapter sequences and low quality bases using the Trim Galore program [70] with default parameters. TopHat 2 was used with default parameters to align reads to their respective genomes [71]. Genome assemblies and annotations were obtained from NCBI, using Galgal5 (annotation release 103) for chicken, Sscrofa10.2 (annotation release 105) for pig, and UMD3.1.1 (annotation release 105) for cattle. No annotation was used during the alignment step to avoid biasing the alignments towards previously annotated splice junctions. Alignments were then filtered with the samtools view ‘-q 15’ parameter to remove those with a MAPQ alignment score of less than 15, which removes low quality alignments and multi-mapped reads. Cufflinks was run on each library individually with the ‘library-type’ parameter set to ‘fr-firststrand’ and with a modified NCBI annotation, containing only the protein-coding genes, provided using the ‘-g’ parameter. Transcriptomes were then combined using Cuffmerge with the NCBI annotation provided using the ‘-g’ parameter to generate a set of transcripts whose expression levels could be measured across tissues [72]. Final expression levels were generated using Cuffnorm with the combined GTF file output by Cuffmerge and with the ‘-library-norm-method’ parameter set to ‘geometric’ and ‘library-type’ parameter set to ‘fr-firststrand’.

### Identification of LncRNAs

Genome annotations from NCBI were used to match assembled transcripts with known genes. As mentioned in the previous section, annotated non-coding transcripts were removed from the annotations by filtering elements that did not have ‘gene_biotype = protein_coding’ so that only protein-coding genes were used to filter assembled transcripts in order to create a completely de novo set of lncRNAs. Any transcript with a Cufflinks class code of “=”, indicating a transcript matching an annotated gene, was removed from the combined set of transcripts. To reduce false positives, mono-exonic transcripts were also omitted, as they are likely to be transcriptional noise. The remaining sequences were then aligned to the Swiss-Prot database [62] to identify homology with known proteins, as well as the Pfam-A database [61] to locate protein domains. Protein sequences were downloaded from their respective websites and NCBI-BLAST [73] was used with the blastx algorithm with default parameters to align translated RNA to the protein databases. Any transcript with a hit in either of these databases with an e-value less than 0.001 was removed, leaving the final set of long non-coding RNAs (lncRNAs). Coding potential scores were calculated for every lncRNA using FEELnc [64] with default parameters. For positive training data, mRNA sequences from the NCBI annotation with “gene_biotype = protein_coding” were used. The negative training data used were the lncRNA sequences from the NONCODEv5 database [63] for the species being analyzed. These scores are shown in Additional files 22, 23 and 24. Note that the coding potential scores were not used in the prediction of the lncRNA, but were calculated and provided as a confidence metric. Overlap of the predicted lncRNA with the NONCODEv5 database was determined using NCBI-BLAST with the blastn command. An evalue cutoff of 1e-5, percentage identity (pident in tabular output parameter) greater than 50%, and query coverage (qcovs in tabular output parameter) greater than 50% was used. All other parameters were default. A few lncRNA were tested with PCR to validate they were not genomic DNA contamination. This is shown in Additional file 25.

### Correlation of expression of lncRNA and nearby protein-coding genes

The correlation between lncRNA and nearby protein-coding genes was calculated using Spearman correlation, which ranks both sets of expression values and calculates the Pearson correlation based on ranks rather than raw expression values. No cutoff value was used and all pairs of lncRNA and protein-coding genes were included in the calculation.

### Tissue-specific LncRNAs identification

Tissue-specific lncRNAs were identified using the tissue specificity index (TSI) [74]. TSI is defined as:

$$ \uptau =\frac{\sum_{i=1}^N\left(1-{x}_i\right)}{N-1} $$

where *N* is the number of tissues and *x*_*i*_ is the expression of the lncRNA *x* in tissue *i* normalized by the maximum expression value. Transcripts with a TSI of greater than 0.9 in both replicates were considered tissue specific. This threshold is recommended in Yanai, et al. [74]. As previously described, Cuffnorm was used to measure expression values, using the “-library-norm-method” parameter set to “geometric”. This uses a normalization method similar to DESeq rather than the default method of calculating FPKM, which is now considered obsolete in favor of TPM. Enriched GO terms were determined using the DAVID Bioinformatics Resource version 6.8 [75, 76] with the default parameters. A *p*-value cutoff of 0.01 was used to consider significant enrichment. The gene list input into DAVID contained every gene from the lncRNA-gene pairs for every lncRNA specific to the tissue. The background was the default set used by DAVID, which is the entire set of genes for the species.

### Conservation of LncRNAs

NCBI BLAST+ 2.2.29 [73] was used to align lncRNA sequences to each other across species. Alignment was done using default parameters as well as using the relaxed parameters “-word_size 7 -reward 1 -penalty -2”. To identify orthologous pairs, a reciprocal method was used, requiring that the best scoring hit (measured by e-value) when aligning species A to species B matched the best scoring hit when aligning the opposite direction, species B to species A. Only alignments with an e-value under the threshold of 10e-5 were used.

OrthoFinder (0.2.8) [77] was used with default arguments to identify groups of orthologs using the NCBI RefSeq proteins for chicken, cattle, pig, human, and mouse. The proteins were then mapped to genes, and only the groups containing at least one gene from all five species (12,390 groups) were kept for further downstream analysis. The classifier function of FEELnc [64] was used to associate lncRNAs with genes within 50,000 bp upstream or downstream, a distance cut-off used in previous studies [78]. LncRNAs from different species that are associated with genes in the same ortholog group are considered putative orthologs. Enriched GO terms were determined using DAVID as described in the previous subsection. To generate multiple sequence alignments of the lncRNAs in the conserved groups, ClustalW (2.1) was used with default parameters [79].

## Additional files

Additional file 1: Sequences of Chicken LncRNAs. A fasta file containing all the lncRNA sequences from chickens. (FA 34950 kb)

Additional file 2: Sequences of Cattle LncRNAs. A fasta file containing all the lncRNA sequences from cattle. (FA 14461 kb)

Additional file 3: Sequences of Pig LncRNAs. A fasta file containing all the lncRNA sequences from pigs. (FA 21227 kb)

Additional file 4: Exon Locations of Chicken LncRNAs. The genomic locations of the exons of all chicken lncRNAs. (GTF 7220 kb)

Additional file 5: Exon Locations of Cattle LncRNAs. The genomic locations of the exons of all cattle lncRNAs. (GTF 4359 kb)

Additional file 6: Exon Locations of Pig LncRNAs. The genomic locations of the exons of all pig lncRNAs. (GTF 9045 kb)

Additional file 7: Mapping to NONCODE. The NONCODE IDs for each lncRNA found in the NONCODE database, as described in the Methods section. For lncRNA that had multiple matches in the NONCODE database, the NONCODE ID for the match with the highest bit score is used. (XLSX 151 kb)

Additional file 8: LncRNA Classes from Chickens. (TSV 3281 kb)

Additional file 9: LncRNA Classes from Cattle. The output from the FEELnc Classifier program, which finds nearby protein-coding genes for each lncRNA and classifies their positional relationship. See Additional file 8 for column descriptions. (TSV 1794 kb)

Additional file 10: LncRNA Classes from Pigs. The output from the FEELnc Classifier program, which finds nearby protein-coding genes for each lncRNA and classifies their positional relationship. See Additional file 8 for column descriptions. (TSV 3182 kb)

Additional file 11: Expression of Chicken LncRNAs. FPKM values for all lncRNAs from each RNA-seq library (2 libraries per tissue) in chickens. (TSV 12559 kb)

Additional file 12: Expression of Cattle LncRNAs. FPKM values for all lncRNAs from each RNA-seq library (2 libraries per tissue) in cattle. (TSV 15586 kb)

Additional file 13: Expression of Pig LncRNAs. FPKM values for all lncRNAs from each RNA-seq library (2 libraries per tissue) in pigs. (TSV 14242 kb)

Additional file 14: Tissue-specific Indices of Chicken LncRNAs. The calculated tissue-specific indices (TSI) for each lncRNA in each tissue. The “TSI A” and “TSI B” columns are the TSI calculated for each biological replicate. The “TSI Both” column is the F1 score of the TSI from both replicates. F1 is calculated as (2 * A * B) / (A + B) where A is TSI A and B is TSI. The “Name” and “Location” columns give the ID and genomic location of the lncRNA, and the following columns are the FPKM values from each library. (XLSX 1551 kb)

Additional file 15: Tissue-specific Indices of Cattle LncRNAs. The calculated tissue-specific indices (TSI) for each lncRNA in each tissue. The “TSI A” and “TSI B” columns are the TSI calculated for each biological replicate. The “TSI Both” column is the F1 score of the TSI from both replicates. F1 is calculated as (2 * A * B) / (A + B) where A is TSI A and B is TSI. The “Name” and “Location” columns give the ID and genomic location of the lncRNA, and the following columns are the FPKM values from each library. (XLSX 1122 kb)

Additional file 16: Tissue-specific Indices of Pig LncRNAs. The calculated tissue-specific indices (TSI) for each lncRNA in each tissue. The “TSI A” and “TSI B” columns are the TSI calculated for each biological replicate. The “TSI Both” column is the F1 score of the TSI from both replicates. F1 is calculated as (2 * A * B) / (A + B) where A is TSI A and B is TSI. The “Name” and “Location” columns give the ID and genomic location of the lncRNA, and the following columns are the FPKM values from each library. (XLSX 1754 kb)

Additional file 17: GO terms associate with tissue-specific lncRNAs in chickens. (XLSX 149 kb)

Additional file 18: GO terms associate with tissue-specific lncRNAs in cattle. This file contains tables from the DAVID analysis tool for each of the eight tissues, showing GO terms enriched by genes associated with tissue-specific lncRNAs in cattle. GO terms outside a significance cutoff of *p*-value < 0.01 are shaded in red. See Additional file 17 for column descriptions. (XLSX 171 kb)

Additional file 19: GO terms associate with tissue-specific lncRNAs in pigs. This file contains tables from the DAVID analysis tool for each of the eight tissues, showing GO terms enriched by genes associated with tissue-specific lncRNAs in pigs. GO terms outside a significance cutoff of *p*-value < 0.01 are shaded in red. See Additional file 17 for column descriptions. (XLSX 144 kb)

Additional file 20: Conserved LncRNAs. The 39 groups of orthologous genes across all five species are listed with the associated lncRNAs. Human and mouse lncRNA IDs are NCBI transcript IDs. (XLSX 21 kb)

Additional file 21: Multiple Sequence Alignments for Conserved LncRNAs. A multiple sequence alignment file generated by ClustalW is included for each of the 39 groups of lncRNAs associated with orthologous genes across all five species. (ZIP 445 kb)

Additional file 22: FEELnc Coding Potential Scores for Chicken LncRNAs. The coding potential scores calculated by FEELnc. The “coding_potential” column is the coding potential score, with 0 being the least likely to be coding and 1 being most likely. The “label” column is 0 or 1 to indicate if the score lies above or below the cutoff determined by FEELnc using cross validation. 0 indicates a predicted lncRNA while 1 is a predicted coding transcript. (XLSX 217 kb)

Additional file 23: FEELnc Coding Potential Scores for Cattle LncRNAs. The coding potential scores calculated by FEELnc. The “coding_potential” column is the coding potential score, with 0 being the least likely to be coding and 1 being most likely. The “label” column is 0 or 1 to indicate if the score lies above or below the cutoff determined by FEELnc using cross validation. 0 indicates a predicted lncRNA while 1 is a predicted coding transcript. (XLSX 173 kb)

Additional file 24: FEELnc Coding Potential Scores for Pig LncRNAs. The coding potential scores calculated by FEELnc. The “coding_potential” column is the coding potential score, with 0 being the least likely to be coding and 1 being most likely. The “label” column is 0 or 1 to indicate if the score lies above or below the cutoff determined by FEELnc using cross validation. 0 indicates a predicted lncRNA while 1 is a predicted coding transcript. (XLSX 323 kb)

Additional file 25: RT-PCR Gel Images for Validation. Gel images from a few RT-PCRs to verify a few of the predicted lncRNAs. (PPTX 203 kb)
