# Supplementary material for: Genome-wide identification of tissue-specific long non-coding RNA in three farm animal species
Source: BMC Genomics. 2018 Sep 18;19:684. doi: 10.1186/s12864-018-5037-7 (PMC6145346; doi:10.1186/s12864-018-5037-7)
Supplement: Supplementary file 25 — RT-PCR Gel Images for Validation. Gel images from a few RT-PCRs to verify a few of the predicted lncRNAs. (PPTX 203 kb) [file 12864_2018_5037_MOESM25_ESM.pptx]

## Slide 1
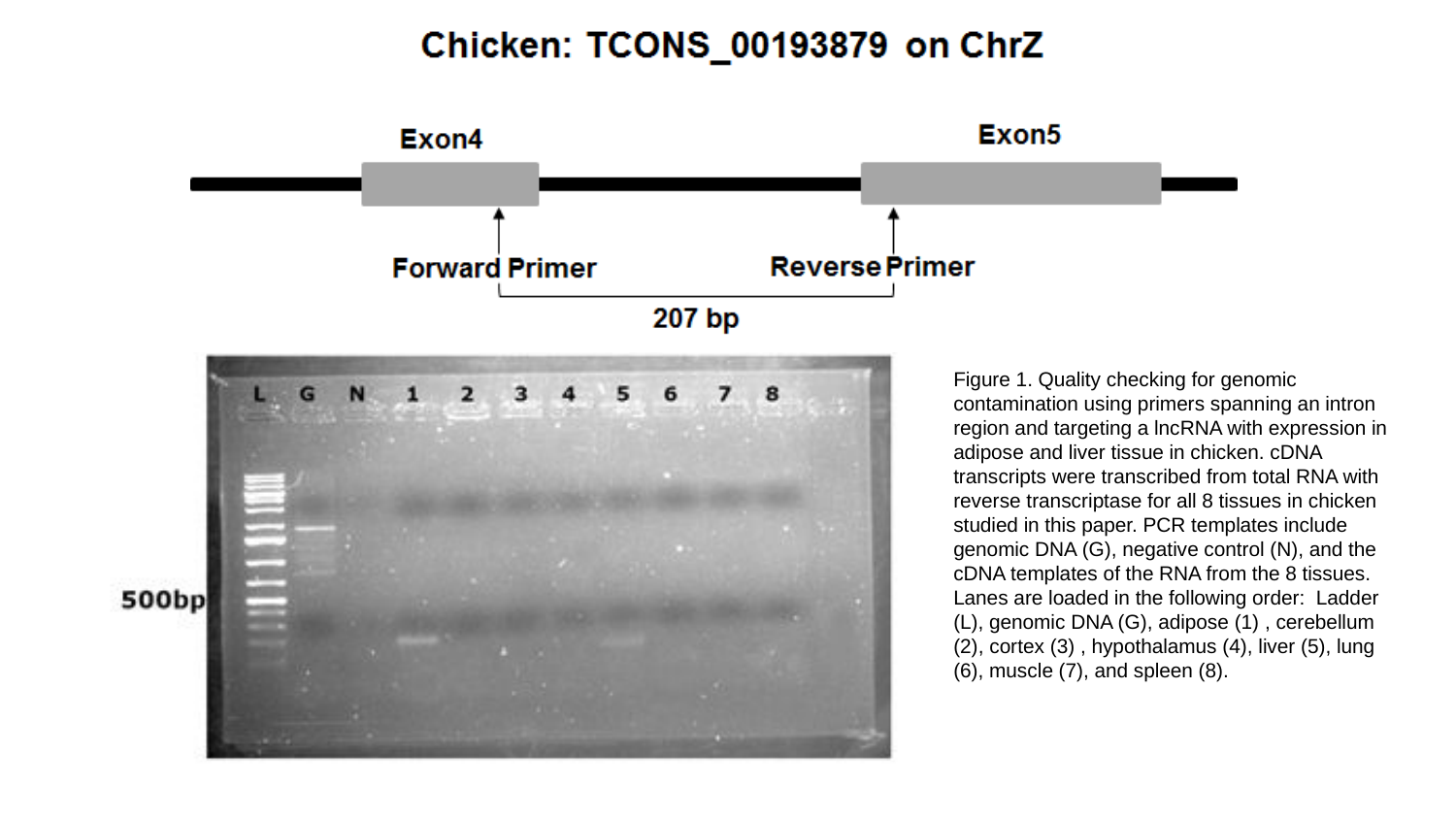

Figure 1. Quality checking for genomic contamination using primers spanning an intron region and targeting a lncRNA with expression in adipose and liver tissue in chicken. cDNA transcripts were transcribed from total RNA with reverse transcriptase for all 8 tissues in chicken studied in this paper. PCR templates include genomic DNA (G), negative control (N), and the cDNA templates of the RNA from the 8 tissues. Lanes are loaded in the following order: Ladder (L), genomic DNA (G), adipose (1) , cerebellum (2), cortex (3) , hypothalamus (4), liver (5), lung (6), muscle (7), and spleen (8).

## Slide 2
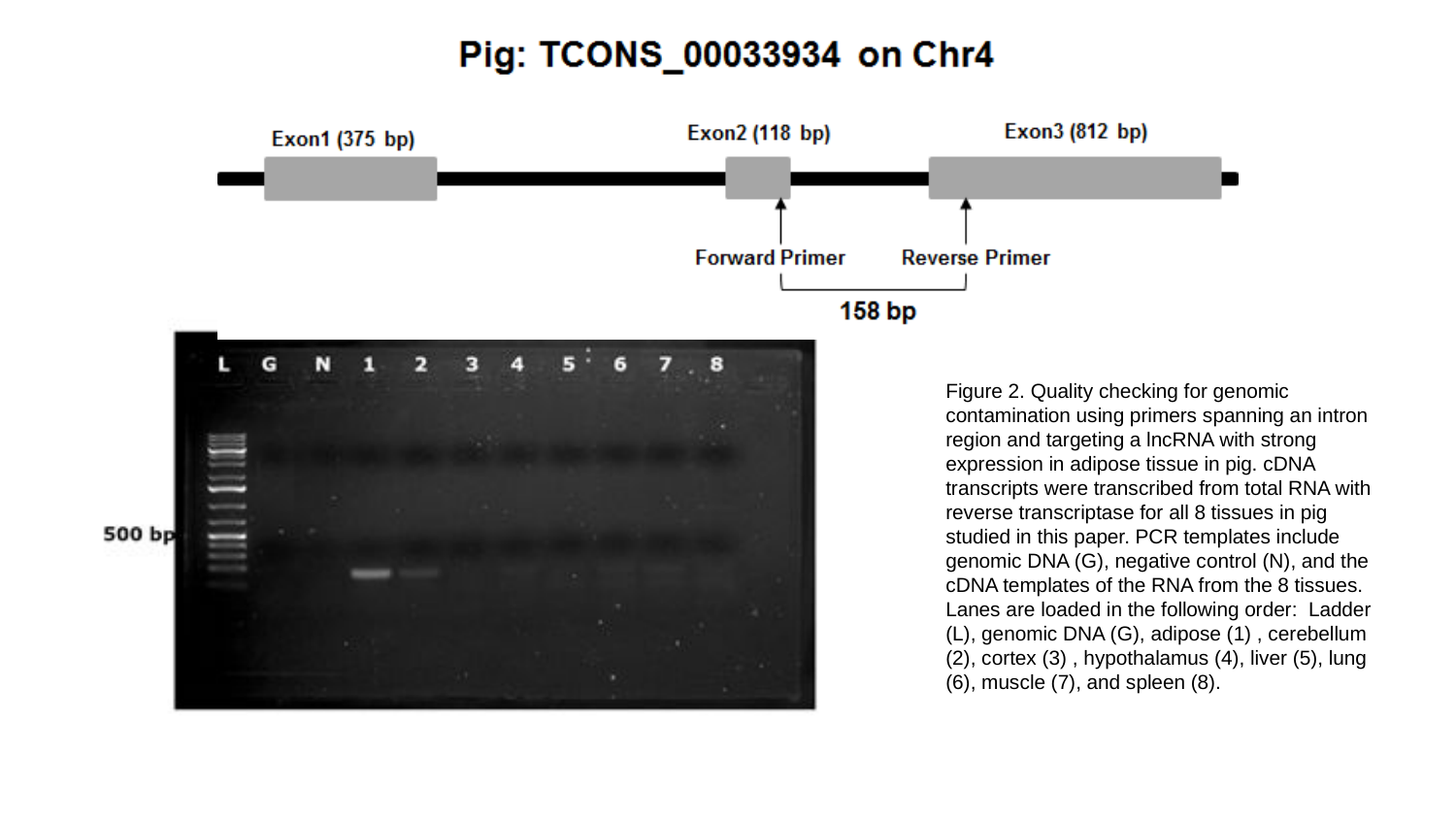

Figure 2. Quality checking for genomic contamination using primers spanning an intron region and targeting a lncRNA with strong expression in adipose tissue in pig. cDNA transcripts were transcribed from total RNA with reverse transcriptase for all 8 tissues in pig studied in this paper. PCR templates include genomic DNA (G), negative control (N), and the cDNA templates of the RNA from the 8 tissues. Lanes are loaded in the following order: Ladder (L), genomic DNA (G), adipose (1) , cerebellum (2), cortex (3) , hypothalamus (4), liver (5), lung (6), muscle (7), and spleen (8).

## Slide 3
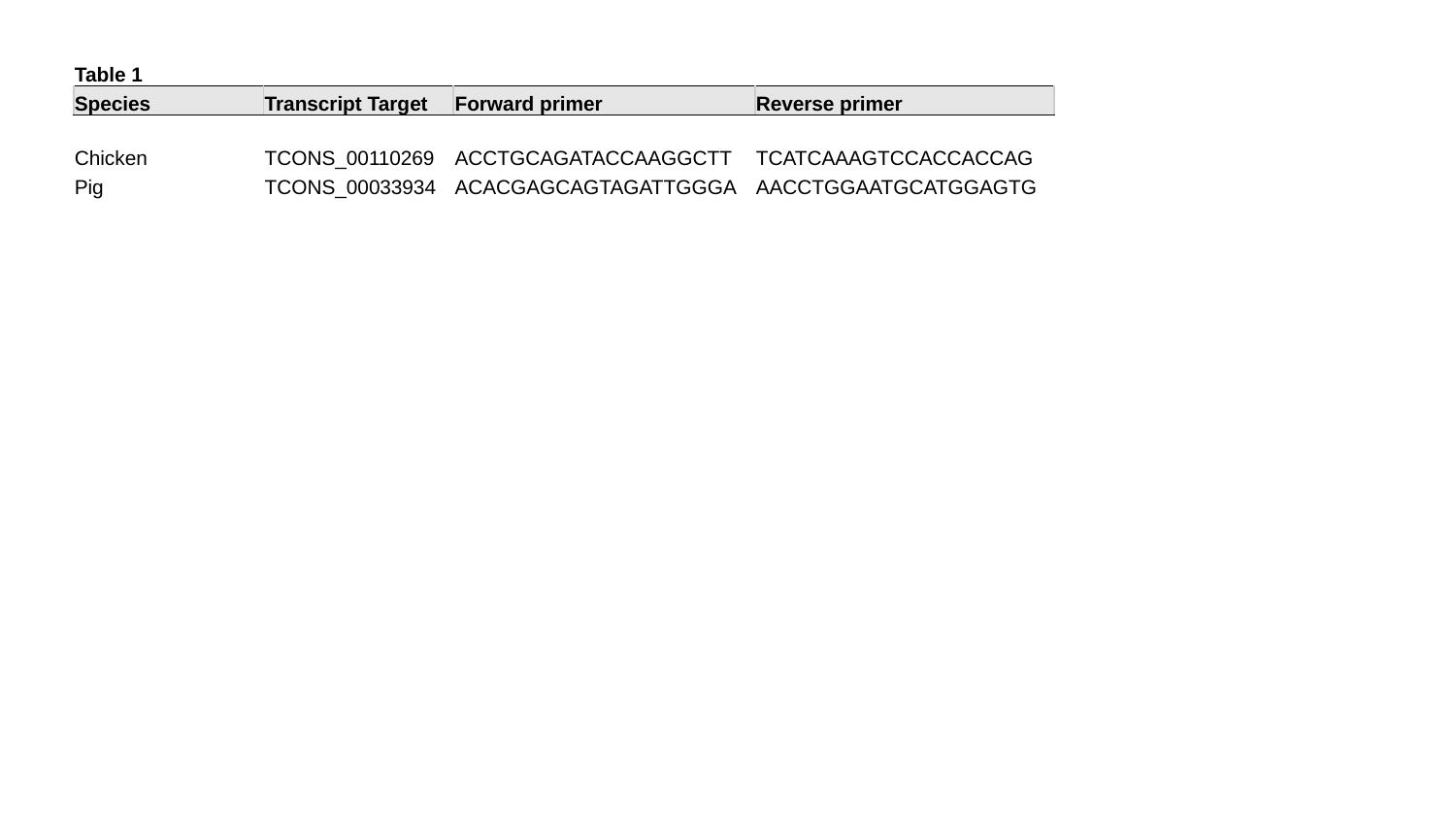

| Table 1 | | | |
| --- | --- | --- | --- |
| Species | Transcript Target | Forward primer | Reverse primer |
| Chicken | TCONS\_00110269 | ACCTGCAGATACCAAGGCTT | TCATCAAAGTCCACCACCAG |
| Pig | TCONS\_00033934 | ACACGAGCAGTAGATTGGGA | AACCTGGAATGCATGGAGTG |
